# Moyamoya syndrome in a 6-year-old β-thalassemia major patient: A case report

**DOI:** 10.1016/j.radcr.2025.04.129

**Published:** 2025-05-29

**Authors:** Maryam Shaukat, Muhammad Aman, Hania Fatima, Faheemullah Khan, Naila Nadeem, Sadaf Altaf, Khabab Abbasher Hussien Mohamed Ahmed

**Affiliations:** a Medical College, The Aga Khan University, Karachi 74200, Pakistan; b Department of Radiology, The Aga Khan University Hospital, Karachi 74200, Pakistan; c Department of Radiology, Cleveland Clinic, Cleveland, OH, USA; d Department of Oncology, The Aga Khan University Hospital, Karachi 74200, Pakistan; e Faculty of Medicine, University of Khartoum, Khartoum 11111, Sudan

**Keywords:** Moyamoya disease, Beta-thalassemia, Cerebral infarction, Pediatrics, Stroke

## Abstract

Moyamoya disease (MMD) is a rare, chronic cerebrovascular disorder involving progressive bilateral stenosis of the internal carotid artery and its branches, forming abnormal collateral networks that appear as a “puff of smoke” on angiography. **While the term MMD is used when the condition is idiopathic, it is called moyamoya syndrome (MMS) when associated with underlying systemic conditions**. Reports of MMS with β-thalassemia major are rare, with fewer than 6 cases documented; herein, we report a case of a 6-year-old female, a known case of β-thalassemia major, who exhibited left-sided hemiparesis, revealing radiological and clinical features consistent with MMS. MRI, CTA, EEG, and Echocardiography were used as diagnostic workups to confirm the diagnosis. The patient was treated with hyper-transfusion therapy and pharmacological management, with an ongoing consideration of surgical intervention. The rarity of the occurrence of moyamoya syndrome in β-thalassemia major patients has been discussed, with emphasis on the importance of early recognition and intervention to mitigate neurological complications. This case contributes to the limited literature on β-thalassemia major and MMS occurring together, underscoring the need for heightened clinical awareness and timely management strategies.

## Introduction

Moyamoya disease (MMD) is a rare chronic cerebrovascular disease marked by progressive bilateral steno-occlusive changes in the terminal segment of the internal carotid artery (ICA) and/or the proximal portion of its branches, anterior cerebral arteries (ACA) and middle cerebral arteries (MCA). These vascular changes are accompanied by the formation of abnormal compensatory collateral networks at the base of the brain, resembling hazy puff of smoke, otherwise known as moyamoya in Japanese, on angiography [1].

While MMD is an idiopathic and predominately genetically triggered vasculopathy, it is termed moyamoya syndrome (MMS) in the presence of underlying systemic conditions such as neurofibromatosis type I and II, down syndrome, cranial radiation, meningitis and hemoglobinopathies, particularly sickle cell anemia [2,3]. However, literature pertaining to the incidence of MMS alongside β-thalassemia major continues to represent a rare occurrence, with less than 6 cases documented in the literature, as concluded using search engines like PubMed and Google Scholar [[4], [5], [6], [7], [8]]. Herein, we report a case of a 6-year-old female with a known history of β-thalassemia major, who came to the outpatient department with symptoms of left-sided hemiparesis.

## Case presentation

A 6-year-old female, previously diagnosed with β-thalassemia major, presented to the hematology outpatient clinic accompanied by her parents, reporting an acute onset of left-sided weakness.

On examination, the patient demonstrated mild pallor indicative of anemia, without jaundice or digital clubbing. Abdominal assessment revealed a soft, nontender abdomen with no hepatosplenomegaly. Vital signs were recorded as follows: temperature of 37°C, blood pressure of 119/56 mm Hg, pulse rate of 110 beats per minute, and respiratory rate of 24 breaths per minute. Notably, she had not undergone blood transfusions; her ongoing medications included folic acid, thalidomide, hist-op, and ascard. Neurologically, the patient was alert and responsive, with a left-sided motor power graded at 4/5 and mild facial asymmetry. The remainder of her general, neurological, and cardiovascular examinations were unremarkable. Her immunization and birth history were normal except spontaneous vaginal delivery at 34 weeks gestation.

The patient’s recent complete blood count (CBC) revealed a hemoglobin level of 9.2 g/dL (reference range: 10-14 g/dL), hematocrit of 30.2% (34.0-40.0), red cell distribution width (RDW) of 31.1% (12.1-16.9), and a platelet count of 591 × 10⁹/L (200-450 × 10⁹/L). Hemoglobin electrophoresis by high-performance liquid chromatography (HPLC) showed elevated HbF (96.9%), normal HbA₂ (3.1%), and absence of HbA. A peripheral blood smear indicated microcytic hypochromic anemia with anisocytosis, poikilocytosis, and polychromasia, consistent with β-thalassemia.

Further history from the guardians disclosed recurrent seizures over the past 3 years, with recent symptoms of slurred speech and difficulty walking following a left-sided stroke 15 days prior. The patient also reported headaches and vertigo persisting for 2 months. An MRI scan conducted at an outside facility identified a subacute ischemic infarct in the left occipital lobe and an attenuated posterior cerebral artery (PCA). Based on these findings, a hyper-transfusion protocol was initiated, and repeat MRI scans were recommended.

**Fig. 1:**
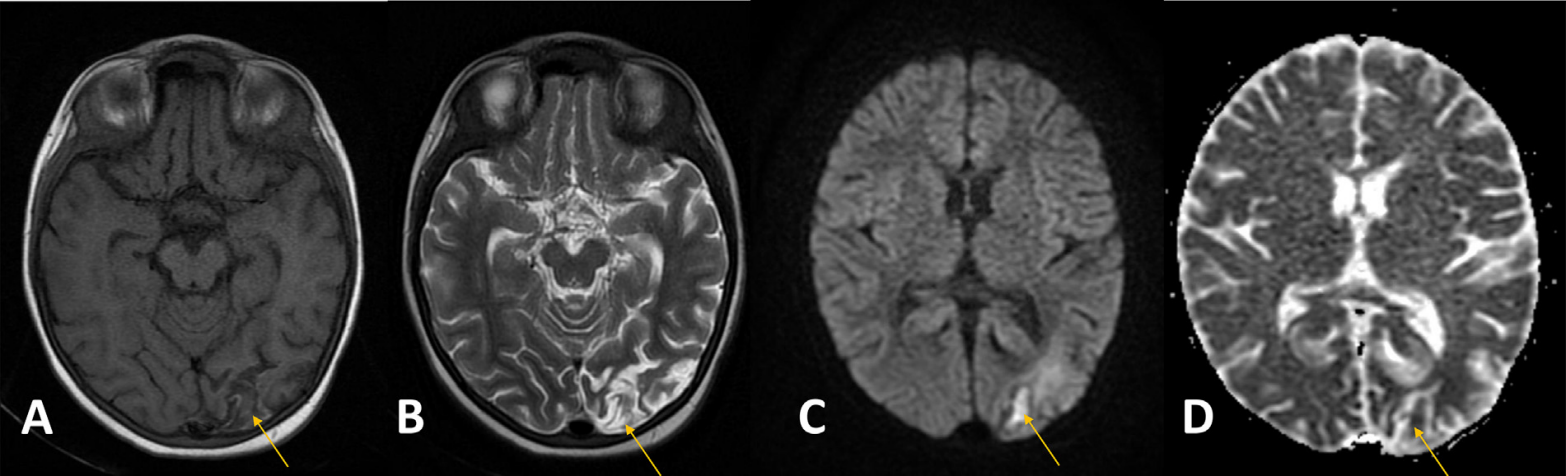
T1 (A), T2 (B), DWI (C) and ADC (D), T1 Iso-hypointense, T2 iso-hyperintense area in the left occipital cortex (arrow) demonstrating diffusion restriction corresponding to ADC mapping representing acute infarct. T1 GYRAL hyperintensity is also seen in the left occipital lobe, representing laminar necrosis secondary to a prior event of ischemia (arrowhead).

**Fig. 2:**
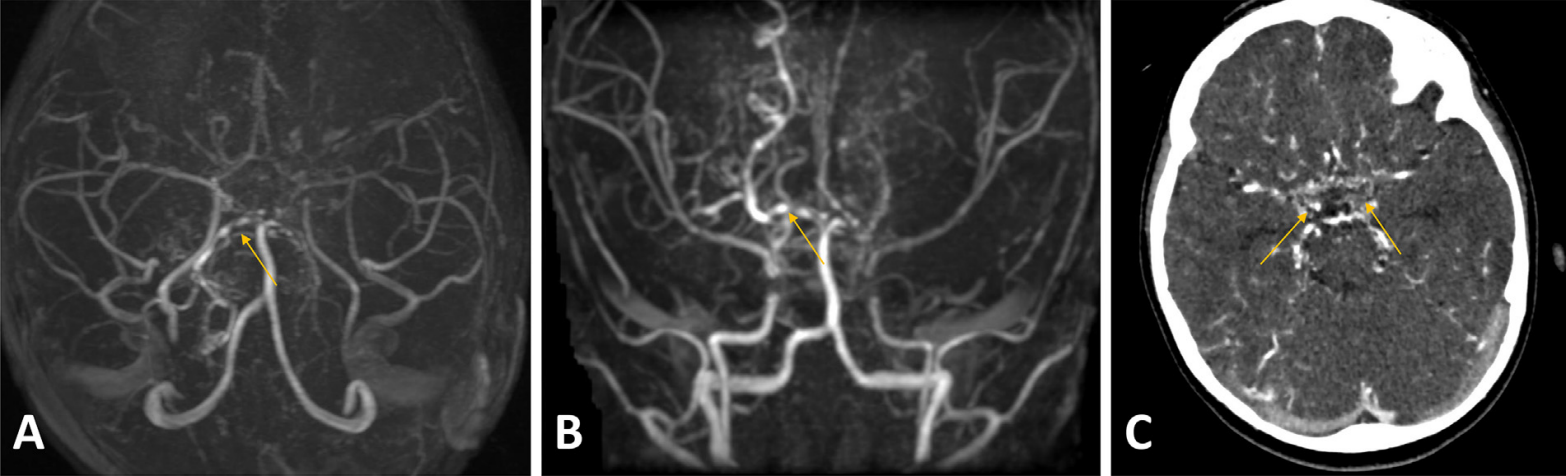
MRA 3D axial (A) and coronal (B), beaded appearance (arrowheads) of the circle of Willis with the formation of numerous collaterals (CTA, C arrows) demonstrating puff of smoke appearance.

**Fig. 3:**
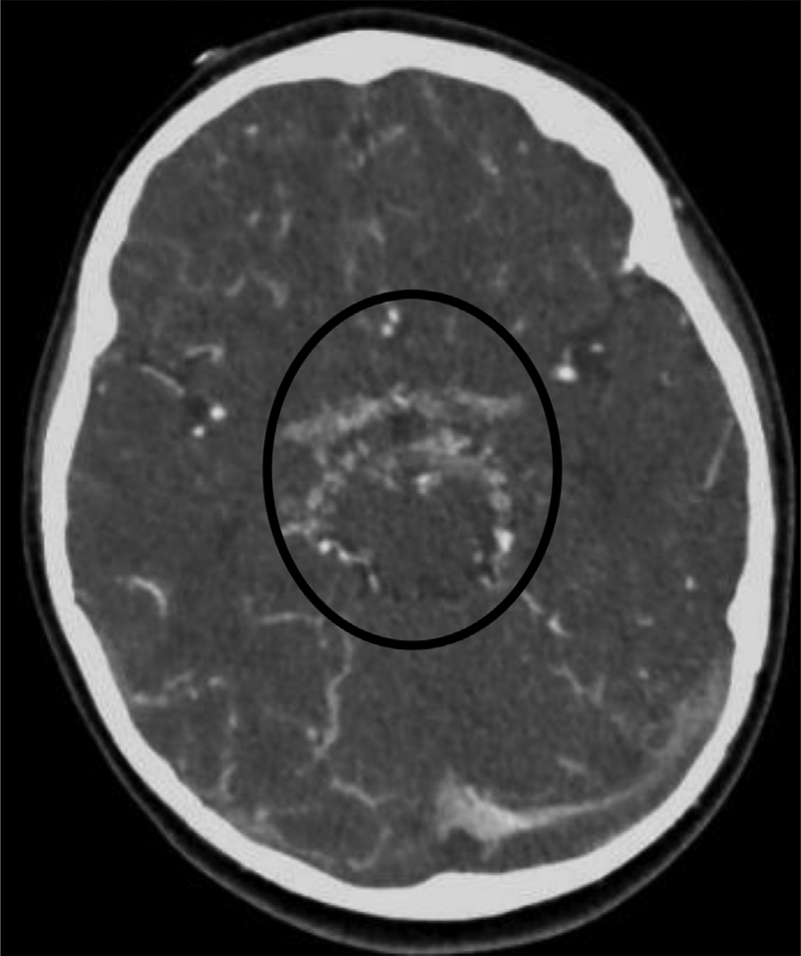
Formation of numerous collateral (circle).

Following a transfusion of irradiated packed red cells, MRI reassessment at our institution revealed multifocal subcortical and periventricular ischemic changes, an acute infarct with postischemic hyperperfusion, and adjacent cortical laminar necrosis in the left occipital lobe (Fig. 1). Additional findings included significantly attenuated, irregular, and beaded intracranial vessels with multifocal narrowing and collaterals, suggestive of moyamoya phenomenon. For confirmation, computed tomography angiography (CTA) with intravenous contrast was performed, demonstrating a beaded appearance of the bilateral ICA and MCA, especially in the Circle of Willis region, with extensive tortuous collateral formation (Fig. 2, Fig. 3). Similar patterns were noted in the bilateral lenticulostriate and thalamoperforating vessels, consistent with stage III MMD per the Suzuki Staging System [9]. This stage involves progressive ICA stenosis with prominent collateral networks, particularly lenticulostriate and thalamoperforating vessels, compensating for reduced blood flow but increasing the risk of ischemic events.

**Fig. 4:**
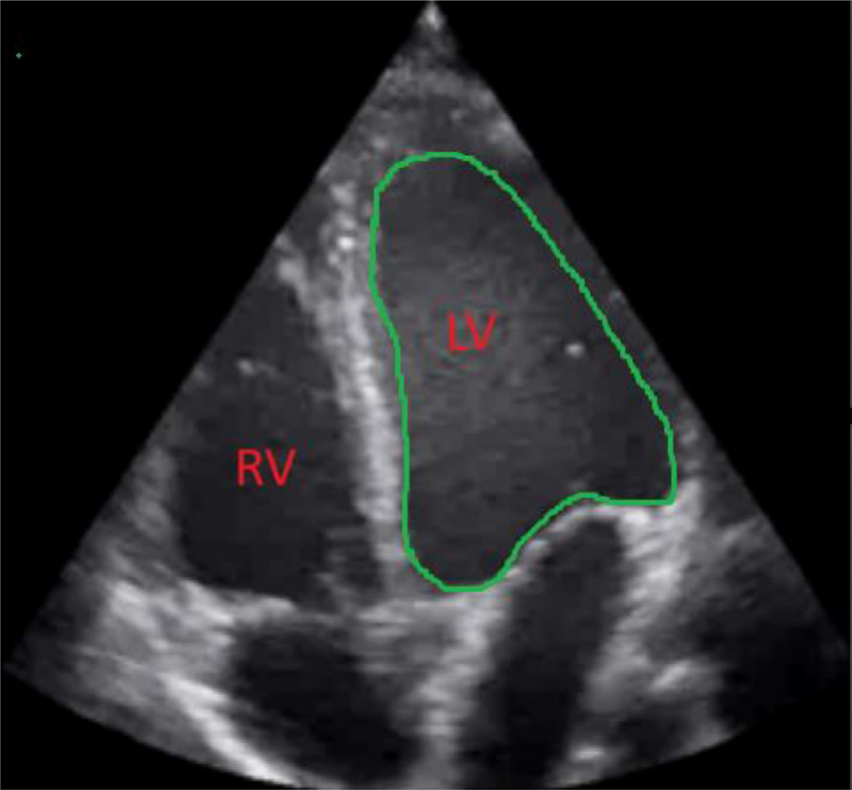
TTE Echo shows mildly dilated left ventricle.

**Fig. 5:**
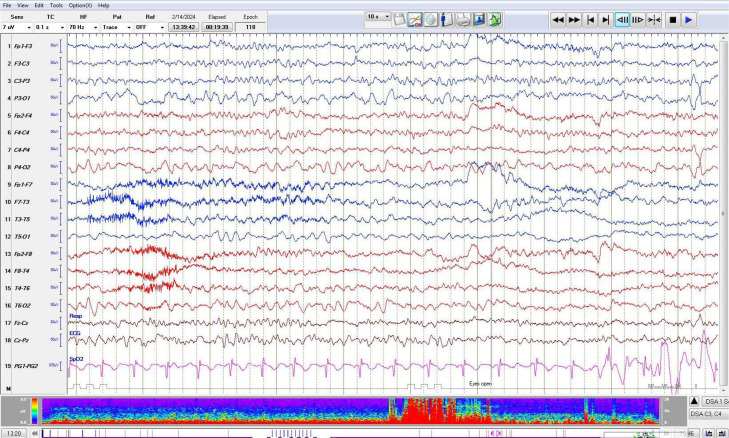
Abnormal EEG in view of slow posterior dominant rhythm in theta range, findings are suggestive of mild encephalopathy.

Considering clinical and radiological findings, a diagnosis of MMS was established. To exclude secondary causes of stroke and evaluate seizure activity, pediatric transthoracic echocardiography (T4TE) and electroencephalography (EEG) were conducted. TTE showed a mildly dilated left ventricle with mildly reduced systolic function (Fig. 4). EEG revealed no epileptiform activity but showed a slow posterior dominant rhythm in the theta range (6-7 Hz), suggestive of mild encephalopathy (Fig. 5).

Since diagnosis, the patient has been managed conservatively with monthly blood transfusions, along with the addition of levetiracetam and pyridoxine hydrochloride to her medication regimen. Follow-up evaluations over the past 6 months have shown no new neurological deficits. Hemoglobin levels have remained stable, albeit slightly low, fluctuating between 7 and 9 g/dL. The patient’s guardians are currently considering bone marrow transplantation as a potential option to improve symptom management. A referral to the neurosurgery department has been made for further evaluation and consideration of surgical intervention.

## Discussion

The incidence of MMD varies significantly worldwide, with higher prevalence in Asia than in Europe, Africa, and the Americas [10]. Although more than 25 cases have reported an association between β-thalassemia and MMS, only 5 cases have thus far reported an occurrence of β-thalassemia major alongside MMS [[4], [5], [6], [7], [8]]. Other commonly reported variations include β-thalassemia intermedia and hemoglobin E β-thalassemia. In Pakistan, however, pediatric cases of MMD are scarcely documented, with only a few reports published to date. To our knowledge, our case is the sixth reported instance of these 2 conditions co-occurring and the first 1 documented in Pakistan. MMD commonly affects females and demonstrates a bimodal age distribution, typically presenting in the first and fourth decades of life [[10], [11], [12]], as seen in this case.

The underlying pathophysiology of MMD suggests that the chronic stenosis of the internal carotid arteries and proximal segments of the middle and anterior cerebral arteries reduces cerebral blood flow, leading to collateral formation through lenticulostriate and thalamoperforating arteries. This longterm cerebral ischemia induces overexpression of proangiogenic factors, resulting in the formation of fragile collateral capillaries, visualized as a “puff of smoke” on angiography. This network compensates for the blood supply to the basal ganglia and thalamus [13]. Microscopically, affected vessels display fibrocellular intimal thickening, leading to arterial stenosis and medial thinning [14]. Gene mutations in RNF213, BRCC3/MTCP1, and GUCY1A3 have been documented in MMD patients, with a positive familial history observed in 10%-15% of cases [11,15].

Although MMD may be asymptomatic, it typically presents with cerebral ischemic events, including transient ischemic attacks (TIA), ischemic infarcts, intracranial hemorrhages, and epilepsy [1,11]. While ischemia is the predominant presentation in pediatric patients, adult cases often manifest with hemorrhagic MMD. Symptoms associated with compromised cerebral blood flow include speech disturbances, weakness, loss of consciousness, headaches, aphasia, hemiparesis, and seizures [12].

In MMS, seizures, especially those involving PCA territories, are concerning. Ischemic injury in these areas contributes to neurological deficits, including visual impairment, and heightens seizure risk due to neuronal dysfunction and the formation of epileptogenic foci [16]. The progressive nature of MMS, with recurrent ischemic events, exacerbates these risks, impacting the quality of life significantly.

Concurrent hematological conditions, such as sickle cell disease, hereditary spherocytosis, β-thalassemia, Fanconi anemia, and combined hemoglobinopathies, may expedite the disease progression [17,18]. In our case, β-thalassemia appears as a potential etiological factor for MMS. The absence of β-globin subunits promotes the expression of pro-coagulant phospholipids (e.g., phosphatidylethanolamine, phosphatidylserine) in red blood cells, resulting in a hypercoagulable state, particularly in patients who have not received transfusions, as with this 6-year-old patient. This state predisposes patients to thromboembolism, early atherosclerosis, and platelet aggregation [19,20]. β-thalassemia’s hypercoagulable state, marked by progressive intracranial artery stenosis, predisposes to thrombotic events and cerebral infarction [21]. Risk factors for increased thrombosis include family history, age >35 years, severe anemia, previous splenectomy, infrequent transfusions, and serum ferritin levels ≥1000 mg/L [22]. Additionally, ineffective erythropoiesis and hemolysis contribute to tissue hypoxia, endothelial hypertrophy, and microvascular stenosis [19]. Chronic anemia is thus a significant risk factor for MMS [20,23].

Initially classified as nontransfusion-dependent thalassemia (NTDT), this patient later required transfusions for disease management. Despite limited evidence supporting transfusion efficacy in MMD, the patient exhibited neurological improvement following her first transfusion, though hemoglobin levels remained low [20,24]. Literature suggests that by maintaining hemoglobin ≥9 g/dL through transfusions thrombotic risk can be reduced [24]. Thus, timely transfusions might have mitigated the neurological complications observed in our case.

These targeted pretransfusion hemoglobin levels (9.5-10.5 g/dL) aim to alleviate anemia, suppress ineffective erythropoiesis, and manage iron overload [25]. In our patient, persistent anemia has prompted consideration of hematopoietic stem cell transplantation (HSCT), the only curative option for thalassemia [26]. The patient’s absence of pretransplant risk factors—such as hepatosplenomegaly, liver fibrosis, and cardiac or hepatic iron overload—favors a >90% disease-free survival rate [27]. Thus, addressing the primary thalassemia may improve the prognosis for MMS. However elevated serum ferritin levels correlate with worse outcomes in stroke patients, hinting at a link between high ferritin levels and stroke severity [28]. In this case, however, ferritin data were unavailable, limiting further assessment of this association.

Initial antiplatelet therapy with aspirin has provided symptomatic relief but has limited impact on MMS’s underlying pathophysiology. While antiplatelet agents reduce hemorrhagic stroke risk, they are less effective in preventing ischemic events or enhancing patient autonomy [29]. Adjunctive anticoagulation and thrombolysis may support MMD or MMS treatment; however, surgical revascularization remains the first-line therapy to improve cerebral perfusion and prevent further ischemic events [30]. A retrospective study of 22 patients showed a significant reduction in stroke incidence, from 3.19 to 0.13 strokes per year, postsurgical intervention [31]. Consequently, the neurosurgery department at our institution is considering both direct and indirect revascularization strategies.

## Conclusion

This case underscores the therapeutic advantages of integrating transfusion therapy and prompt anemia management in hemoglobinopathies, notably β-thalassemia, to mitigate the risk of developing MMS. It also emphasizes the necessity of including MMS in the differential diagnosis when evaluating neurological deficits in patients with beta-thalassemia.

## Data availability statement

The data supporting the findings of this study are available from the corresponding author upon reasonable request.

## Ethical approval

As this is a case report, an ethical review was not necessary.

## Patient consent

As this is a case report, an ethical review was not necessary. A written informed consent was obtained from the parents to publish this case report and all imaging findings. The data presented of the patient is kept anonymous.

## CRediT authorship contribution statement

**Maryam Shaukat:** Conceptualization, Data curation, Investigation, Writing – original draft, Writing – review & editing. **Muhammad Aman:** Conceptualization, Supervision, Validation, Writing – review & editing. **Hania Fatima:** Writing – review & editing. **Faheemullah Khan:** Supervision, Validation, Writing – review & editing. **Naila Nadeem:** Supervision. **Sadaf Altaf:** Writing – review & editing. **Khabab Abbasher Hussien Mohamed Ahmed:** Supervision, Writing – review & editing.
